# The impact of aging on morphometric changes in the cerebellum: A voxel-based morphometry study

**DOI:** 10.3389/fnagi.2023.1078448

**Published:** 2023-01-20

**Authors:** Johannes Stalter, Vinuya Yogeswaran, Wolfgang Vogel, Peter Sörös, Christian Mathys, Karsten Witt

**Affiliations:** ^1^Department of Neurology, Carl von Ossietzky University Oldenburg, Oldenburg, Germany; ^2^Center of Neurosensory Sciences, Carl von Ossietzky University Oldenburg, Oldenburg, Germany; ^3^Institute of Radiology and Neuroradiology, Evangelical Hospital Oldenburg, Oldenburg, Germany

**Keywords:** VBM, cerebellum, morphometric changes, MRI, aging

## Abstract

**Introduction:**

Aging influences the morphology of the central nervous system. While several previous studies focused on morphometric changes of the supratentorial parts, investigations on age-related cerebellar changes are rare. The literature concerning the morphological changes in the cerebellum is heterogenous depending (i) on the methods used (cerebellar analysis in the context of a whole brain analysis or specific methods for a cerebellar analysis), (ii) the life span that was investigated, and (iii) the analytic approach (i.e., using linear or non-linear methods).

**Methods:**

We fill this research gap by investigating age-dependent cerebellar changes in the aging process occurring before the age of 70 in healthy participants, using non-linear methods and the spatially unbiased infratentorial template (SUIT) toolbox which is specifically developed to examine the cerebellum. Furthermore, to derive an overview of the possible behavioral correlates, we relate our findings to functional maps of the cerebellum. Twenty-four older participants (mean age 64.42 years, SD ± 4.8) and 25 younger participants (mean age 24.6 years, SD ± 2.14) were scanned using a 3 T-MRI, and the resulting data were processed using a SUIT.

**Results:**

Gray matter (GM) volume loss was found in older participants in three clusters in the right cerebellar region, namely crus I/II and lobule VI related to the frontoparietal network, with crus I being functionally related to the default-mode network and lobule VI extending into vermis VIIa related to the ventral-attention-network.

**Discussion:**

Our results underline an age-related decline in GM volume in the right cerebellar regions that are functionally predominantly related to non-motor networks and cognitive tasks regions of the cerebellum before the age of 70.

## 1. Introduction

Age-related changes, such as the decline in cognitive function and difficulties in motor tasks, have their origin in the central nervous system (CNS; Demnitz et al., 2017). As a part of the CNS, the cerebellum plays a well-known and important role in balance and motor activities (Albus, 1971; Ito, 2002; Koziol et al., 2014). In recent years, it has been shown that the cerebellum can be divided into functional subregions. Additionally, various studies have highlighted its key role in non-motor tasks (Schmahmann and Sherman, 1998; Buckner, 2013; Guell et al., 2018b; Diedrichsen et al., 2019). Research on the role of the cerebellum in functional networks demonstrated the impact of the cerebellum on motor and non-motor tasks (Hoche et al., 2018; Guell et al., 2018b; King et al., 2019). These results, combined with task-activation imaging, led to the development of a map of task-related activations corresponding to their functional networks (Guell et al., 2018a). In summary, and as Bernard and Seidler (2014) point out in their review, recent literature has shown that the cerebellum is involved in various functions that are important for everyday functioning (Bernard and Seidler, 2014). Given the fact that the cerebellum shows a volumetric decline in the aging process to an extent that is known from other cerebral areas such as the prefrontal cortex, it is important to investigate the impact of aging on the cerebellum to better understand the role of the cerebellum in general and the process of aging itself, including its contributing factors (Bernard and Seidler, 2014; Gellersen et al., 2021).

This leads to the question concerning the role of the cerebellum in the process of aging. Although there are different methods to investigate morphometric changes, most of our understanding of age-related volumetric changes in the brain is derived from studies using voxel-based morphometry (VBM). With VBM, local alterations of brain tissue from magnetic resonance imaging (MRI) scans can be compared. In the first decade of the use of VBM, MRI scanners were often not able to cover the entire brain correctly, thereby neglecting the infratentorial parts of the brain (Diedrichsen, 2006). Apart from the problem of spatial resolution, an additional challenge was related to the lacking ability of pipelines to process the fine anatomical details of the cerebellum. As a result, early VBM applications often did not analyze the brainstem and the cerebellum in their entirety.

However, even with more sophisticated techniques, inconsistent findings are reported across different studies regarding age-related cerebellar gray matter loss (Torvik and Torp, 1986; Oguro et al., 1998; Luft et al., 1999; Xu et al., 2000; Raz et al., 2001; Tamnes et al., 2013; Hulst et al., 2015). This inconsistency in the results is partly due to biological factors as well as technical and methodological issues, whereby a further aspect is the type of statistical analysis, i.e., using linear or non-linear analytical approaches. However, in general, the findings suggest a loss of gray matter with a tendency to the posterior lobe. An overview of the results of previous studies and the possible biological and analytical biases is provided in Supplementary Table S1. Taken together, there is a lack of additional information concerning the way the gray matter of certain lobules is impacted by age.

Romero postulated a relative plateau phase of the cerebellar volume between 25 and 65 years and a rapid change afterward (Romero et al., 2021). Therefore, it is important to examine the whole lifespan and additional periods before and after 70 years separately so that the effects of changes in the timespans do not overlap and complementary information can be added. While most previous studies investigated linear changes and included age groups over 80 years (Hulst et al., 2015; Yu et al., 2017; Filip et al., 2019), recent studies demonstrate a non-linear age-related change in cerebral volume and gray matter volume, thereby questioning the applicability of linear methods (Ziegler et al., 2012; Ramanoël et al., 2018). To our knowledge, only two research articles describe an investigation of the morphometric changes in the cerebellum during the midlife aging process with a non-linear volume-based lobule-dependent approach (Bernard and Seidler, 2013; Bernard et al., 2015). Only one of these studies used a middle-aged group, while the other compared two groups, one older and one younger. These studies illustrate the importance of further investigation using a voxel-wise VBM approach to examine a lobule-independent pattern of cerebellar atrophy.

To address these issues, we used a 3.0 Tesla MRI scanner and the relatively new spatially unbiased infratentorial template (SUIT) toolbox to improve infratentorial voxel-wise analysis in younger and older participants. This technique improves the spatial resolution and therefore allows a more detailed analysis of the cerebellum. Bernard and Seidler highlight the importance of looking closely at the subregions of the cerebellum since different regions are affected unequally (Bernard and Seidler, 2014). With our study, we aim to fill the research gap concerning the exploration of age-dependent cerebellar morphometric changes (i) in a healthy population younger than 70 by comparing two defined groups that are 40 years apart in age, using (ii) non-linear statistics and (iii) analytic tools specifically developed to explore cerebellar data. In addition, we aim to correlate these findings to previously described networks and task-related cerebellar maps. Given the existing findings in the literature, we hypothesized that the older group would have atrophies that are primarily bihemispheric in the posterior lobe and crus I.

## 2. Materials and methods

### 2.1. Participants

Participants were enrolled according to the following inclusion criteria: right-handedness (the Edinburgh Handedness Inventory-Short form includes four items ranging between −100 and +100, whereby right-handedness is defined as a score higher than 60; Veale, 2014), no medical history of neurological or psychiatric disease, and no use of psychoactive drugs. As exclusion criteria, we screened for contraindications for the use of MRI, severe depression, meaning a Beck’s Depression Inventory (BDI) sum score >20 (higher scores indicate more severe symptoms of depression) and a decline in cognitive status, measured with the Montreal Cognitive Assessment (MoCA) and defined as a sum score greater 26 points on a scale ranging from 0 to 30 (larger scores indicate better cognitive functioning; Beck et al., 1961; Nasreddine et al., 2005). The years of education were collected using a self-reporting questionnaire, whereby school years and years of vocational training were summarized. For the younger group, we included participants aged 18–30 years while the older group comprised participants aged 55–69 years. All participants signed the written informed consent for this study, which was approved by the local ethics committee. The study was registered in the German register of clinical studies (Deutsches Register Klinischer Studien) with the number DRKS00015727.

### 2.2. MRI scan acquisition

Structural imaging was acquired in the neuroimaging unit of the Carl von Ossietzky University Oldenburg with a 3.0 Tesla MRI System (Siemens Magnetom Prisma Hardware Configuration, Syngo MR E11 Software Configuration; Siemens Medical Solutions, Erlangen, Germany) equipped with a 64-channel head–neck-coil. We used a T1-weighted Magnetization Prepared Rapid Gradient Echo Sequence (MPRAGE; number of slices = 224; echo time TE = 255 ms; repetition time TR = 2,000 ms; flip angle = 9°; field of view = 240 × 240 mm; acquisition matrix = 320 × 320; voxel size = 0.8 × 0.8 × 0.8). In addition to the MPRAGE sequence, we performed a T2-weighted scan and a fluid-attenuated inversion recovery (FLAIR) sequence, to look for pathological findings in each participant. Each scan was evaluated by a neuroradiologist (CM).

### 2.3. MRI data processing

The preprocessing of the MRI scans was conducted using the following main steps. First, the T1-weighted scans were manually oriented to the same origin, namely the anterior commissure. The cerebellar brain tissue was isolated in all scans and transformed into segmentation maps by the SUIT toolbox [Diedrichsen, 2006; Spatially unbiased atlas template of the cerebellum and brainstem (RRID:SCR_004969)]. The SUIT toolbox uses the SPM12 algorithm for the segmentation process, and only the gray matter segmented images were used for subsequent steps. We chose a posterior probability value of 0.2 for tissues to be infratentorial. Secondly, the generated maps were checked manually for any remaining extratentorial tissue. During this step, no extratentorial tissue was found. To gain better alignment between the subjects, we normalized the produced images to the SUIT template by using non-linear deformation. The SUIT template improves the alignment of the fissures and reduces the spatial spread compared to the Montreal Neurological Institute (MNI) template (Diedrichsen, 2006). Finally, we smoothed the images with an 8 mm full-width half-maximum Gaussian kernel to match the Gaussian distribution assumption for the statistical analysis. We separately calculated the total intracranial volume (TIV) with the computational anatomy toolbox (CAT 12) (RRID:SCR_019184) for SPM12 and used these values as covariates in the subsequent analysis (Gaser et al., 2022, BioRxiv).

We performed a two-sample *t*-test of the voxel value for the volume using sex and TIV as covariates to compare both groups using SPM12 [https://www.fil.ion.ucl.ac.uk; SPM (RRID:SCR_007037)]. The resulting statistical parametric map identified the cerebellar regions with significant differences in the cerebellar volume with a primary threshold of *p* < 0.001 and a cluster size of *k* > 100. Subsequently, we performed a stricter analysis with a family-wise error correction for multiple comparisons (*p* < 0.05) and a voxel threshold of *k* > 300 for the cluster to focus on contiguous atrophies. In this way, by comparing two groups, namely a younger and an older cohort, we investigated the non-linear changes that may occur between early and late adulthood. In the final step, we used the Anatomy Toolbox for SPM12 [Forschungszentrum Jülich GmbH; SPM Anatomy Toolbox (RRID:SCR_013273)] to match significant clusters with the anatomical probability map (Eickhoff et al., 2005, 2006, 2007).

To correlate the morphological findings with functional regions of the cerebellum and motor and non-motor networks of the cerebellum, we used the *neurosynth.org* database.^1^ This meta-analysis platform scans openly available scientific articles on the internet for (f)MRI coordinates and anatomical or activation-related terms related to these coordinates in the articles. With this information, it builds a database that correlates single voxels with cognitive domains or functional connectivity (z-score). According to the platform, it consists of 51 studies with cerebellar coordinates that are used for the analysis.

### 2.4. Statistics

We checked the demographic and psychological assessment data for its distribution using the Kolmogorov–Smirnov test. Since the data of the psychological assessment were not normally distributed, we used the Mann–Whitney-*U* test to compare the results of the BDI and MoCA between the two groups. To detect the effect size of the significant difference in the BDI, we used an eta-square. Due to the sample size being smaller than 50, the non-parametric Mann–Whitney-*U* test was also used for the demographic variables while the distribution of the genders was analyzed using a chi^2^ test.

## 3. Results

### 3.1. Participant characteristics

The younger group includes 25 right-handed healthy participants (13 female, 12 male; mean age ± standard deviation [SD]: 24.60 ± 2.14 years; range 20–29 years) and the older group includes 24 right-handed healthy participants (11 female, 13 male; mean age ± standard deviation [SD]: 64.42 ± 4.80 years; range 55–69 years). One of the female participants in the older group was excluded due to the incompletion of the MRI scan. The MRI scans were evaluated by a clinical neuroradiologist (CM) to exclude the presence of incidental findings (i.e., findings leading to further diagnostics in clinical practice) that would serve as an exclusion criterion. The number of years of education is nearly the same in both groups, with a mean of 17.70 years (±2.10 years SD, range 14–22 years) in the younger and 17.54 years (±3.94 years SD, range 12–29 years) in the older group (*p* = 0.659; *z* = −0.442). Regarding the BDI analysis, the older group displays a mean score of 3.79 (span 0 to 11) whereas the data from the younger group yielded a mean score of 2.16 (span 0 to 10) resulting in a significant difference between the two groups (*p* = 0.007; *z* = −2.681). With an effect size of **η**^**2**^ = 0.014, the effect is rather small. The mean scores for the MoCA are quite similar, with 28.33 SD 1.2 vs. 28.96 SD 1.2 (older vs. younger), respectively, showing no significant difference between the two groups (*p* = 0.085, *z* = −0.1.721). The demographic and psychological data are shown in Table 1.

**Table 1:** Demographic characteristics of the younger and the older participants.

	Older group	Younger group	Mann–Whitney-*U* Test or Chi-square Test
Sample size	*N* = 24	*N* = 25	
Male/female	13/11	12/13	*p* = 0.132
Age mean (SD, years)	64.42 (4.80)	24.60 (2.14)	*p* = 0.00; *z* = −6.075
Age median (years)	66	24	
BDI mean (SD)	3.79 (3.00)	2.16 (3.23)	*p* = 0.007; *z* = −2.681
MoCA mean (SD)	28.33 (1.27)	28.96 (1.20)	*p* = 0.085; *z* = −1.721
Education in years mean (SD)	17.54 (3.94)	17.7 (2.100)	*p* = 0.659; *z* = −0.442

SD, standard deviation; BDI, Beck’s Depression Inventory; MoCA, Montreal Cognitive Assessment, data are given as mean and standard deviation (SD).

### 3.2. VBM

**Figure 1:**
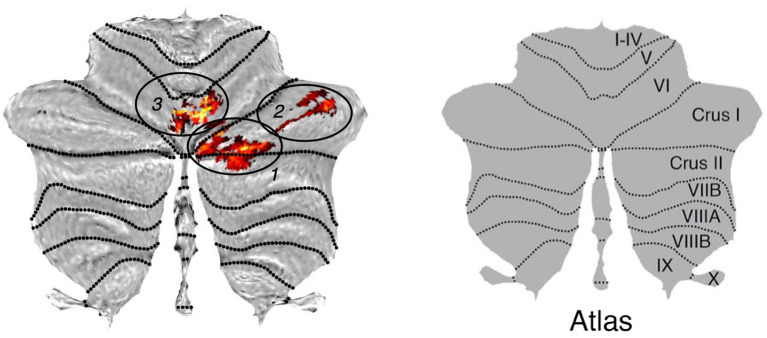
Cluster of the SUIT analysis. Max. t-value is 5.37. Note that the part of cluster one, located in lobule VIIB is not visible in this presentation of the results. The right figure is adopted from Guell et al. (2018a,b).

The analysis identified three clusters with a significant loss of gray matter in the older participants (Table 2). Figure 1 displays the cluster on a cerebellar flatmap, using the plotting function of the SUIT toolbox (Diedrichsen and Zotow, 2015). The largest cluster (790 Voxel; p_FWE-corr_ = 0.000; *T* = 6.98) overlapped with 41.5% in the right lobule VIIa crus I, with 22.0% in the right lobule VIIa crus II, and 5.3% in the right lobule VI as well as 2.9% in the right lobule VIIb. It is important to note that the part in lobule VIIb is not visible in the figures. The second cluster (338 Voxel; p_FWE-corr_ = 0.000; *T* = 6.69) is 100.0% located in the right lobule VIIa crus I while the last cluster (319 Voxel; p_FWE-corr_ = 0.000; *T* = 7.04) demonstrated 18.8% of its voxels in the right lobule VI, 17.9% in the lobule VIIa of the right vermis, 15.7% in lobule VIIa of the left vermis, and 4.1% in lobule VI of the right vermis. The missing parts to make up 100% could not be assigned with sufficient certainty by the toolbox.

**Table 2:** Cluster of the SUIT analysis with size and localization of the cluster assigned to the MNI space.

Cluster	*p*_FWE-corr_	Cluster size in (voxel)	*T*	*x*	*y*	*z* {mm}
3	0.00	319	7.04	5	-66	−25
			18.8% right Lobule VI 17.9% right Lobule VIIa Vermis 15.7% left Lobule VIIa Vermis			
1	0.00	790	6.89	25	−81	−32
			41.5% right Lobule VIIa crus I 22.0% right Lobule VIIa crusII 5.3% right Lobule VI 2.9% right Lobule VIIb			
2	0.00	338	6.69	51	−59	−30
			100% right Lobule VIIa crus I			

*p*_FWE-corr_, *p*-value family-wise error corrected.

### 3.3. Assignment to functional data

**Figure 2:**
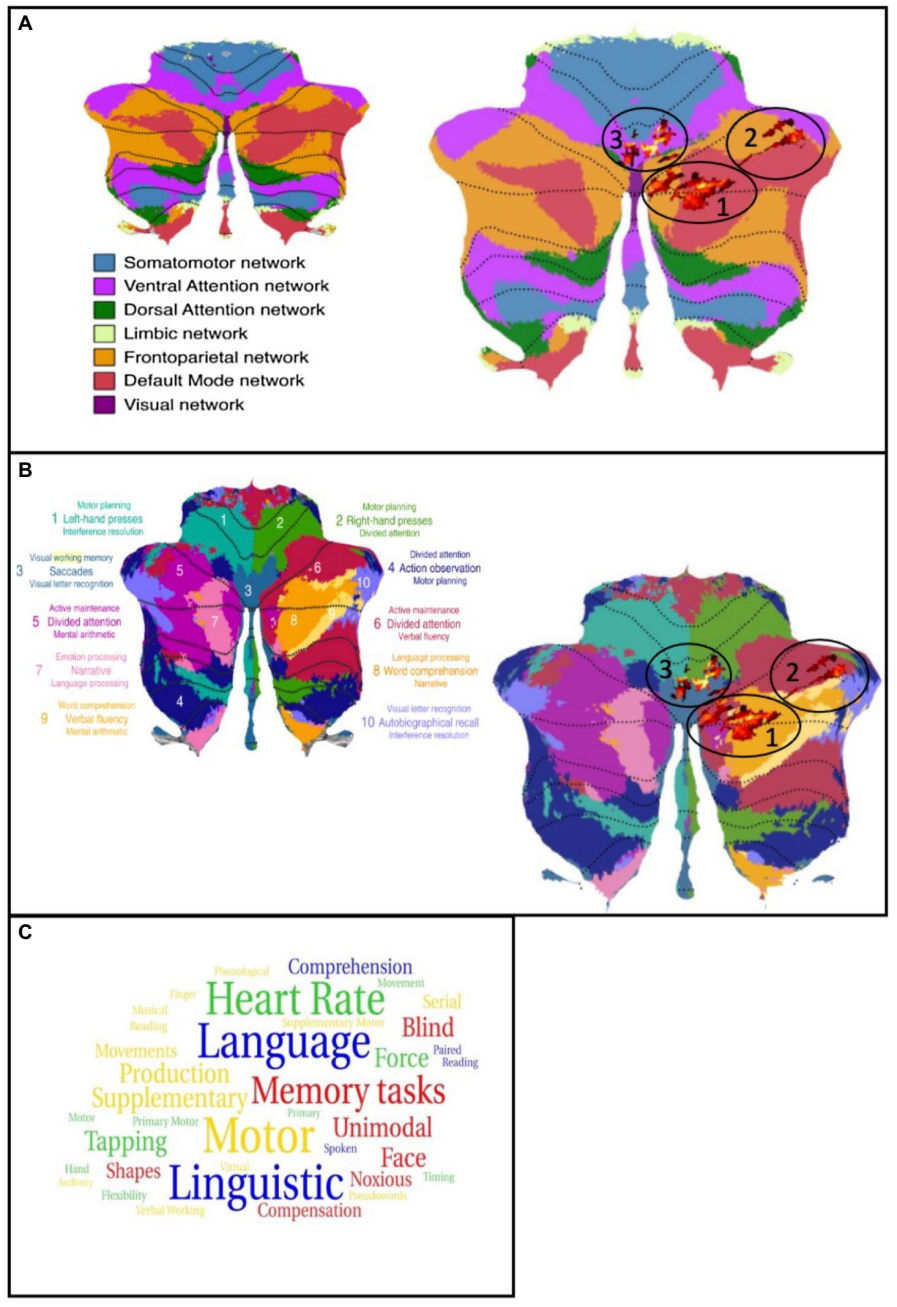
**(A)** left: Map of the functional networks (Buckner et al., 2011); Right: Cluster of the analysis plotted on the map of functional networks, atrophies are shown as red colored cluster (circled). **(B)** left: Map of the multi-domain task battery (King et al., 2019); Right: Cluster of the analysis plotted on the map of MDTB, atrophies are shown as red colored cluster (circled). **(C)** word cloud of the neurosynth.org terms. Size of the words is related to the z-score. Blue = cluster 1, red = cluster 2, green/yellow = cluster 3.

In the next step, we matched the cluster found in the present study with the functional network maps reported by Buckner et al. (2011). The first cluster is primarily located in the frontoparietal network while, in contrast, the parts in crus I are located in the default mode network (DMN). The second cluster has its location in the default mode and the frontoparietal network, with few parts found in the ventral attention network. The third cluster is partly detected in the ventral attention network as well as in the somatomotor network. Taking the multi-domain task battery (MDTB) map into account, the clusters are mainly found in regions for visual working memory, attention tasks, and language processing, including language processing, word comprehension, visual working memory, and letter recognition as well as divided attention, maintenance of attention, and verbal fluency. Only one part of one cluster is located in areas relating to motor tasks. These results are shown in Figures 2A,B, displayed on the functional network map of Buckner et al. (2011) (A) and the multi-domain task battery (MDTB) parcellation of King et al. (2019) (B) (Buckner et al., 2011; King et al., 2019). To provide an in-study validation, the coordinates of the three cluster maxima reported in Table 1 were looked up in the neurosynth.org database. Figure 2C shows a word cloud of the terms found in the neurosynth.org database, whereby the size of the words is related to the z-scores. In addition, the results of the analysis with the neurosynth.org database and the maxima of our clusters are demonstrated in the Supplementary materials (Supplementary Table S2). The z-score is the association of the term with the voxel in the whole meta-analysis of the database. For the maximum of cluster 1, corresponding to the functional network, the highest correlation is found for terms related to language processing while the maximum of cluster 2 relates to terms for memory and visual functions. For the last cluster, we analyzed two maxima because of their large spatial expansion, and both are dominated by terms of motor activation, whereby the first one also shows a match with the auditory network.

## 4. Discussion

This study aimed to identify morphological changes in the cerebellar volume that occur between early and later adulthood in a range from 25 years to 65 years by comparing two groups (one younger and one older) of participants. Our literature analysis (see Supplementary material) revealed that results focusing on the age-related cerebellar morphometric changes are driven by (at least) biological factors such as age and the analytical pipeline used, e.g., linear or non-linear approaches as well as tools designed to analyze the whole brain or tools specifically designed to analyze the cerebellum. This study fills the gap in the literature by reporting age-related cerebellar atrophy in two groups of younger, respectively, older healthy participants in a non-linear way using a data analysis pipeline that is developed to assess the cerebellum in detail. Furthermore, we correlated the regions of interest with well-known task-specific regions and network regions.

Using the SUIT toolbox to analyze the difference between the younger and the older group, we found three clusters of atrophy in the cerebellum of the older adults. The largest was mainly found in the right lobule VIIa crus I/II while another cluster was exclusively located in the right lobule VIIa crus I. The third cluster showed voxels in the right lobule VI and the right vermis VIIa. All clusters were limited to cortical regions in the cerebellum. Most of the clusters showed parts in the frontoparietal network and the DMN as well as in the ventral attention network. In contrast, fewer voxels were located in the somatomotor network. Regarding the task activity regions, the voxels were mainly in the working memory and language processing areas. Corresponding to the network analysis, only a small proportion was located in a region that is activated during motor tasks. The activation analysis with *neurosynth.org* showed a high congruence between the activation terms and the function we assumed based on the atrophy cluster. In conclusion, two independent analyses revealed that the patterns of cerebellar atrophy of the compared groups mainly affect areas known to be involved in cognitive processes regarding language and working memory. Without the expected difference in mean age, there were no significant differences for years of education, sex, or the MoCA results. The older group showed a significantly higher score in the BDI compared to the younger group. However, with a median score of 3.79 resp. 2.16, and since the cut-off score for even a mild depression is 13 and no individual was above this score, we considered the difference to be reflecting an unspecific and age-related increase in the physiological BDI items, e.g., a lower sexual drive or altered sleep patterns (Endler et al., 1999; Serafini et al., 2019).

Previous studies mostly used a linear approach to investigate volume changes during the aging process and included participants older than 70 years (see Supplementary Table S2 for more information and Figure 1 supp for graphical details). Findings obtained using this approach are mainly focused on the anterior part of the cerebellum, i.e., lobes I–V. Furthermore, they indicate more widespread volume losses compared to non-linear methods, especially when using volume-based calculations. In this study, we used a non-linear approach to investigate morphometric changes on a voxel-wise basis in the aging process compared between two groups (younger and older) before the age of 70. This specific study design contributes valuable new information concerning the impact of the aging process on the cerebellum that occurs before the age of 70 to complement the findings of studies that examined changes after the age of 70 or studies investigating the whole lifespan (Romero et al., 2021).

Almost comparable studies such as that of Filip et al. reported a bilateral cluster of atrophy in crus I and in the left crus II, the right lobules VI, and right vermis IX (Filip et al., 2019). However, this study used a linear approach with the SUIT toolbox and included older participants for their analysis. A relatively new study comparing fairly small groups of 14 younger and 16 older participants reported a loss of gray matter in the right crus I, a cluster that is also reported in the present study (Ramanoël et al., 2018). The study in question also included older participants, making it challenging to use their results for examining changes that occur before the age of 70. Bernhard and Seidler found a significant volume decline in the anterior lobe as well as in crus I but used a volume-dependent approach which results in more widespread atrophy patterns (Bernard and Seidler, 2013). In their study, the analysis is designed to identify region-related volumetric changes that do not enable reporting on specific brain regions affected by an age-related volumetric change. Taken together, the results of the most comparable studies indicate that the regions consistently affected by gray matter loss are the right crus I/II and the right lobule VI. Other regions, for instance, the anterior lobe or left hemispheric parts, are only partially found. A potential explanation for *in vivo* MRI results showing additional, i.e., new, atrophies in the crus region in more recent studies might be the enhanced ability to detect even more subtle differences due to advanced technological approaches in modern MRI scanners and data processing pipelines. These results are supported by two studies. Firstly, a study by Yu and collegues calculated the velocity of volume loss in the cerebellum, and in their linear model the authors showed the highest rates of volume decline per decade in the right cerebellar hemisphere, in particular in the vermis crus I, the right lobule V, and the right crus I/II (−4.59% vs. −4.28% vs. −3.51% vs. −3.56% per decade), thereby matching our findings of clusters in these anatomical regions (Yu et al., 2017). These regions may be especially prone due to multi-factorial mechanisms consisting of a greater exposure to toxins or toxin vulnerability, changes in the vascular system, and other functional factors (Hulst et al., 2015). An interesting study by Gellersen and colleagues conducted a meta-analysis of atrophy clusters in 13 studies investigating the cerebellum in the elderly (Gellersen et al., 2021) and identified clusters in both hemispheres of the cerebellum, whereby the right-sided cluster was similarly located to the cluster we identified. Furthermore, the authors compared the aging-related cluster with the atrophy cluster found in a meta-analysis of Alzheimer’s disease (AD) patients. Interestingly, these clusters are strictly located in the right hemisphere and thus our results have more of an overlap with an AD atrophy pattern. This specific pattern could be a sign of future cognitive decline, even in healthy elderly people that show no signs of cognitive impairment at present. This is tentatively supported by the idea that single-side atrophy can be (partly) compensated by the functional and structural connections, whereby this theory must be more closely evaluated in future studies. In this context, a study by Guell et al. is worth mentioning, which found different gradients in the cerebellum explaining the heterogeneity of function in anatomically-mirrored regions of the hemispheres (Guell et al., 2018a,b). Unfortunately, no information concerning the demographic details of the studies included in the meta-analysis is available. Concerning the findings of our study, the observation of a clear overlap with an AD pattern of atrophy could be explained by the hypothesis that AD atrophy, in the early stages of the disease, merely aggravates the effects of a normal early aging process. Therefore, the patterns in younger elderly and AD patients are predicted by the general vulnerability of cerebellar structures, which is supported by the reported findings of Yu et al. (2017).

Apart from the morphological results, we also aimed to map the cerebellar loss of gray matter in the elderly to functional networks and mainly found non-motor networks to be affected. Bernard and Seidler demonstrated the correlation between morphological changes and their behavioral correlate and suggested that there is a positive relationship between cognitive function and the volume of crus I but a negative relationship for motor tasks which is explained as a cognitive interference in motor tasks (Bernard and Seidler, 2013). However, it is worth mentioning that the motor tasks in the study by Bernard and Seidler were limited to a balance test. Another explanation might be related to the role of the frontal lobe in cognitive functions. Paul et al. (2009) found no correlation between cerebellar volume and cognitive performance when corrected for frontal lobe volume (Paul et al., 2009). However, in contrast, a study by Koppelmans et al. (2017) showed a positive correlation between regional cerebellar volume and cognitive performance in mental rotation and verbal working memory. They found the anterior lobe and parts of the posterior lobe to be correlated with motor performance and large parts of the posterior lobe to be related to performance in cognitive tasks such as the letter rotation test and the Rey auditory verbal learning test (Koppelmans et al., 2017). Interestingly, a systematic analysis of age-related cerebellar atrophy and the behavioral outcome in motor and non-motor aspects is lacking. However, our analysis supports the observation that cerebellar changes in the elderly are localized in regions that are involved in cognitive processes rather than motor tasks.

Another approach is to map regions of cerebellar atrophy with activation maps recorded by functional MRI (fMRI) or connectivity analysis. A meta-analysis by Stoodley and Schmahmann demonstrated activity in the regions of crus I/II in the language-related tasks (Stoodley and Schmahmann, 2010). Activations in this region were also determined during working memory and performing spatial tasks, as well as divided attention and attention maintenance. Especially the latter tasks imply an active role in executive functions which is in line with the corresponding results of the maps by Buckner et al. (2011) and King et al. (2019). Thus, this part of the cerebellum was defined as the “cognitive cerebellum.” Furthermore, Guell et al. (2018a,b) mentioned the input from Broca area 46 projecting into crus I Guell et al. (2018a,b). This area is thought to support working memory. Cluster 1 of our study is found in regions that are thought to be activated during default mode tasks as the literature shows an age-related decline of network integrity as well as network connectivity of the DMN (Skouras and Scharnowski, 2019; DeSerisy et al., 2021). The DMN is thought to take over when there is no goal-directed activity and provides self-reference introspection (Mak et al., 2017). The atrophy we observed could therefore underpin changes in this network that were identified in previous studies. Some parts of cluster 3 are located in the regions of the ventral attention network. This network is used to control which information will be actively perceived depending on the currently performed task (Suo et al., 2021) and a loss of neurons in this area could lead to damage in circuits which in turn could explain typical deficits during aging such as problems with concentration and the processing of external stimuli (Seeley et al., 2007; Buckner et al., 2011). Cluster 2 is predominantly localized in the frontoparietal network, which controls other neuronal networks and provides flexible cognitive control (Marek and Dosenbach, 2018).

Considering these results together, two different implications can be derived. On the one hand, a variety of structures and networks that are important for executive functions such as social control or attention are affected. These are important for everyday functioning and can be impaired in elderly people which heavily impacts their quality of life. On the other hand, the age-related loss of cerebellar gray matter volume may impair systems that are needed for language processing such as comprehension, speaking, and reading. This is congruent with the results of the network analysis and the terms we found in the neurosynth.org database analysis showing the highest scores for terms related to language processing. This finding is in line with previously reported results that indicate that especially complex language functions are negatively impacted by age, while speech itself is less affected (Yorkston et al., 2010). Within the MDTB map, the clusters were placed in regions for attention, working memory, and language processing which corresponds to previously highlighted studies and our neurosynth.org database analysis, although additional confirmation using functional MRI (fMRI) data in combination with behavioral testing is needed to further decrypt the neurobiological underpinnings of these results.

## 5. Strengths and limitations

All scans were obtained using the same 3.0 Tesla MRI scanner with the same protocol, thereby ensuring high comparability between scans. The scans were checked for pathological findings and artifacts by four different examiners, one of whom is a trained neuroradiologist, and two additional scans were run to find any morphological distractors. Both sample populations differ in terms of an age span of 40 years and were carefully matched to increase the potential to also detect smaller effects. Another strength is the SUIT toolbox we used which is designed to examine the cerebellum (Diedrichsen, 2006). We did not cover genetic (Sønderby et al., 2021; Brouwer et al., 2022), environmental (Nußbaum et al., 2020), and lifestyle (Bittner et al., 2019, 2021; Dekkers et al., 2019) factors that affect whole brain volume and which might also influence cerebellar volume. Nevertheless, by using larger sample groups, an even smaller difference could have been found with this study design. It is important to keep in mind that in this study, we compared structural data with functional networks. To gain more insight and validate the conclusion, further studies should combine these results with more advanced neuropsychological tests and fMRI data. However, our approach to matching the morphological findings and the result of the comparison with the findings of Buckner and King with the neursynth.org database can support our assumptions about behavioral correlates (Buckner et al., 2011; King et al., 2019; Yarkoni, 2020).

## 6. Conclusion

This study reveals that the effect of aging in the age under 70 on the cerebellum leads to gray matter atrophy, primarily in the right lobule VI and right crus I/II. These findings add strong evidence to the hypothesis that the posterior lobe, in particular, crus I and the posterior vermal part, is highly impacted by the aging process of the cerebellum. This atrophy affects regions that are involved in functions such as language processing, motor tasks, DMN, and working memory as well as executive functions, with most of them being significantly reduced in older adults. Hence, our findings highlight the role of regional cerebellar atrophy in healthy aging. Future studies should further analyze the mechanism behind the relationship between cerebellar atrophy and cognitive decline by combining the structural information with fMRI data for the whole brain and a behavioral test battery.

## Data availability statement

The raw data supporting the conclusions of this article will be made available by the authors, without undue reservation.

## Ethics statement

The studies involving human participants were reviewed and approved by Medical Ethical Committee Faculty VI – Medicine and Health Sciences University of Oldenburg. The patients/participants provided their written informed consent to participate in this study.

## Author contributions

JS: conceptualization, data curation, formal analysis, investigation, methodology, project administration, resources, visualization, writing–original draft, and writing–editing. KW: conceptualization, data curation, formal analysis, investigation, methodology, project administration, resources, supervision, validation, visualization, writing–original draft, and review and editing. CM: investigation, validation, visualization, and writing–review and editing. VY: writing–review and editing. WV: writing–review and editing. PS: writing–review and editing. All authors contributed to the article and approved the submitted version.

## Conflict of interest

The authors declare that the research was conducted in the absence of any commercial or financial relationships that could be construed as a potential conflict of interest.

## Publisher’s note

All claims expressed in this article are solely those of the authors and do not necessarily represent those of their affiliated organizations, or those of the publisher, the editors and the reviewers. Any product that may be evaluated in this article, or claim that may be made by its manufacturer, is not guaranteed or endorsed by the publisher.

## Footnotes

^1^ https://neurosynth.org
