# Supplementary material for: The impact of aging on morphometric changes in the cerebellum: A voxel-based morphometry study
Source: Front Aging Neurosci. 2023 Jan 20;15:1078448. doi: 10.3389/fnagi.2023.1078448 (PMC9895411; doi:10.3389/fnagi.2023.1078448)
Supplement: Supplementary file 1 [file Data_Sheet_1.PDF]

Supplement Table 1

In this mini-review, we present the findings of previous studies assessing the impact of healthy aging on cerebellar morphometry. The following search items were used for the literature overview search in Pubmed and google scholar: “cerebellum”, “ageing” and “voxel-based morphometry/vbm” whereas all studies reporting the effects of diseases were eliminated. All terms were varied if there were alternative spelling and used as MeSh terms as well as [All fields] terms. The search was conducted on the 8th of august 2022.

Literature review for cerebellar morphometry changes during ageing including the main characteristics of these studies: Year of publication, statistical specifications, pipeline of analysis, number of participants, age of participants, summary of the reported findings.

First author	Year	Cluster size	Programm	Statistical method	Participants (Amount, range [years])	Statistical significance, correction type	Findings
**Koppelmans, V.**	2015	n.a.	Freesurfer	General multivariate linear model	217 (64 – 87; mean age 70,7 ± 5.0)	p=0.05	Global cerebellar grey matter volume declines with age
**Taki, Yasuyuki**	2013	n.a.	SPM8	Multiple linear regression model	381 (21 - 80)	p< 0.05 FWE	Positive correlation with age for the posterior lobe of the cerebellum. negative correlation with age in the anterior lobe of the cerebellum

**Raji, C.**	2009	>100	SPM2/ VBM2	Multiple linear regression model	169 (70 – 89)	FDR <0.05	Atrophy in the whole cerebellum, no further explanation
**Yu, Teresa**	2017	n.a.	SUIT/ SPM8	Linear regression Model	479 (7-86)	p< 0.05 FWE	left and right crus, the vermis and in the left lobule VI bialatteraly. Smaller clusters were also seen bilaterally in lobule VIIIa and right lobule I_IV and V. Vermis Crus I displayed the greatest GM loss of the cerebellum with a rate of loss of 4.59 %/decade.
**Hulst, Thomas**	2015	n.a.	SUIT/ SPM12	Voxel-by-voxel correlation for age	334 (18 - 96)		lobules I–V; lobule VI // Crus I/II and lobule VIIb does not show significant volume reduction with age // lobule IX and lobule X with strong degeneration
**Filip, P.**	2019	<200	SUIT	Regression analysis	67 (20 – 76, mean age 45.6 ± 14.6)	p< 0.05 FWE	Bilateral crus I; left crus II; right lobule VI; right lobule IX; left lobule V
**Koppelmans, V.**	2017	n.a.	SUIT/ Freesurfer	Linear regression Model	213 (64 – 87; mean age 70.7 ± 5.1)		Except for bilateral lobule X and right cluster crus II/lobule VIIb, all cerebellar regions showed significantly smaller volumes with older age
**Smith, C.**	2007	n.a.	SPM2	ANCOVA	122 (>55; mean age 75 +/- 7.6)	FDR <0.05	Diffuse reductions of GM volume in the cerebellum. According to the authors no reliable results

**Terribili, D.**	2011	>20	SPM2	General linear model	89 (18-50)	FWE <0.05	negative correlations posterior lobe of the left cerebellum. (-17, -63, -37)
**Bernhard, J**	2013	n.a.	SUIT/ SPM8	Mixed-Model ANOVA	31 (mean age 65 ± 6.4), 23 (mean age 22 ± 3.4)	Bonferroni p<0.002	right lobules I-IV, V, VI, right Crus I, left lobules I-IV, V, VI, left Crus I, left Crus II, vermis lobule VI, and vermis lobule VIIb Right hemisphere was larger
**Bernard, J**	2015	n.a.	SUIT/ SPM8	Regression model	123 (mean age 30.93 ± 13,69)	-	Vermis volume is higher in adolescents, but is smaller in young adults, at which point the downward trend continues, but less rapidly (R2 5 0.253, F(1,121) 5 40.88, P < 0.001). A similar relationship with age was seen in the left and right anterior cerebellum (left: R2 5 0.145, F(1,121) 5 20.45, P < 0.001; right: R2 5 0.164, F(1,121) 5 23.77, P < 0.001). Crus I was fit using a linear model for both hemispheres (left: R2 5 0.171, F(1,121) 5 25.04, P < 0.001; right: R2 5 0.129, F(1,121) 5 17.84, P < 0.001). The relationship for the posterior part between regional volume and age was significantly modeled using a quadratic fit (left: R2 5 0.111, F(2,120) 5 7.46, P 5 0.001; right: R2 5 0.137, F(2,120) 5 9.54, P < 0.001).
**Ramanoël, S.**	2018	>20	SUIT	Two-sample t-test	16 (mean age 40.8± 8.6); 14 (mean age 70.5 ± 6.6)	p < 0.05 FWE corrected	crus I of the right cerebellum 340 mm^3

**Ziegler, G**	2012	>50	SPM8/ VBM8	General linear model	547 (19 - 86)	p<0.0001 FWE	right: V/VI;VII;VIII<br>left: VI/VII
**Good, C.**	2001		SPM99	Two-sample t-test	465 (18 - 79)	p< 0.05 corrected for multiple comparism	Local areas of relative accelerated loss of grey matter volume (i.e., more than the global loss) right cerebellar posterior lobe

Table 1 supp: Abbreviations: SUIT=Spatially unbiased infratentorial template; SPM=Statistical parametric mapping; VBM=Voxel based morphometry; FEW=Family wise error correction; FDR=false discovery rate correction: ANOVA=Analysis of variance; ANCOVA=Analysis of covariance; n.a=not available). Order of the publication according to the age of the participants (before the age of 70 vs after the age of 70), the analytical approaches (linear vs non-linear) and the used processing pipelines (including the whole brain imaging data or specifically develop to assess the cerebellum). Since the methodical approaches were too different to compare, the following studies were excluded from our analysis:

1. Xu, J., Kobayashi, S., Yamaguchi, S., Iijima, K. I., Okada, K., & Yamashita, K. (2000). Gender effects on age-related changes in brain structure. *American Journal of Neuroradiology*, *21*(1), 112–118.
2. Raz, N., Gunning-Dixon, F., Head, D., Williamson, A., & Acker, J. D. (2001). Age and sex differences in the cerebellum and the ventral pons: A prospective MR study of healthy adults. *American Journal of Neuroradiology*.
3. Oguro, H., Okada, K., Yamaguchi, S., & Kobayashi, S. (1998). Sex differences in morphology of the brain stem and cerebellum with normal ageing. *Neuroradiology*, *40*(12), 788–792. https://doi.org/10.1007/s002340050685
4. Luft, A. R., Skalej, M., Schulz, J. B., Welte, D., Kolb, R., Bürk, K., Klockgether, T., & Voight, K. (1999). Patterns of age-related shrinkage in cerebellum and brainstem observed in vivo using three-dimensional MRI volumetry. *Cerebral Cortex*, *9*(7), 712–721. https://doi.org/10.1093/cercor/9.7.712
5. Abe, O., Yamasue, H., Aoki, S., Suga, M., Yamada, H., Kasai, K., Masutani, Y., Kato, N., Kato, N., & Ohtomo, K. (2008). Aging in the CNS: Comparison of gray/white matter volume and diffusion tensor data. *Neurobiology of Aging*, *29*(1), 102–116. https://doi.org/10.1016/j.neurobiolaging.2006.09.003
6. Kalpouzos, G., Chételat, G., Baron, J. C., Landeau, B., Mevel, K., Godeau, C., Barré, L., Constans, J. M., Viader, F., Eustache, F., & Desgranges, B. (2009). Voxel-based mapping of brain gray matter volume and glucose metabolism profiles in normal aging. *Neurobiology of Aging*, *30*(1), 112–124. https://doi.org/10.1016/j.neurobiolaging.2007.05.019

Reasons for exclusion:

1. Scans were printed out and volumes were calculated using the cross section area
2. Volumes were calculated manually using the cross section area
3. Images were obtained using a 0.2 T scanner and the volumes were calculated manually using the cross section area
4. Volumes were calculated manually using the cross section area
5. No further anatomical differentiation were given
6. No further anatomical differentiation were given

The results of a voxel-based morphometry are driven at least by biological factors such as age and the analytical pipeline used e.g., linear or non-linear approach and toolboxes designed to analyze the whole brain or toolboxes specifically designed to analyze the cerebellum. Therefore, in a next step we ordered the reported publications according to three rules (see supple. Figure 1). First, we divided the studies in those who examined participants in different stages of age with a cut-off by an age of 70 years. Background is the observation that older age significantly drives the progression of brain atrophy (Romero et al., 2021). Therefore, it is reasonable to separately investigate brain atrophy in these two populations. Second, brain atrophy analyses vary according to different statistical models applied to the data e.g., linear or a non-linear analyses. Background of this dichotomy is the recent observation that brain atrophies progress in a non-linear fashion (Bethlehem et al., 2022). Divergent study results might be explained by linear vs. non-linear statistical approaches. Suppl. Figure 1 demonstrates the impact of both analytical ways. Third, whereas the initial toolboxes to measure brain volume were developed to examine supra-tentorial parts of the brain, newer pipelines focus on infra-tentorial brain regions. The supplemental figure 1 demonstrates that one study published by Bernhard and Seidler (2013) examined the impact of age on brain atrophy using a non-linear approach with the SUIT toolbox as an specifically developed toolbox for a morphometric analysis of the cerebellum. However, this study chooses an area depended volumetric analysis and did not perform a voxel-wise comparison between younger an older adults. This specific gap in the literature is the motivation of the present study.

Supplement Figure 1:

Graphical summary of cerebellar atrophies according to the literature in table 1 supp

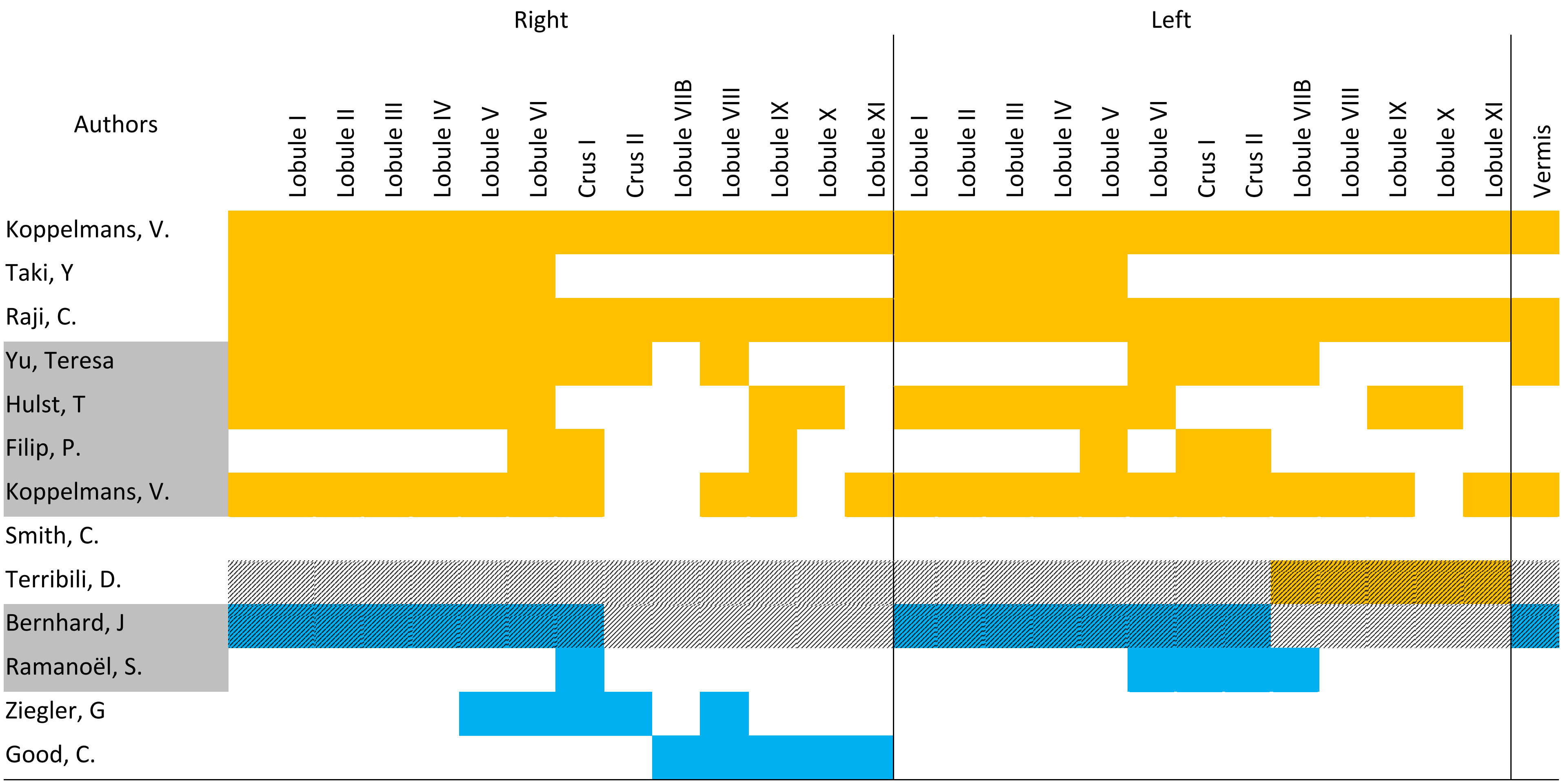

Open – whole brain analysis	Non-shaded lines – studies include a population > 70		Blue – studies used a non-linear statistical approach
Grey – SUIT analysis	Shaded lines – studies excluded a population < 70		Orange – studies used a linear statistical approach

Figure 1 supp: Graphical summary of cerebellar atrophies according to the literature in table 1 supp marked by age (non-shaded lines represent studies including a population over the age of 70 and shaded lines indicate studies that include a younger population), linear or non-linear statistical analysis (in orange studies using a linear method and in blue studies using a non- linear method) and the tool box used for data analysis (white first column used analytic pipelines that assess whole brain volume and gray first column represent studies using stool boxes specifically develop to analyze the cerebellum).

Supplemental Table 2

Results of the analysis of the neurosynth.org-database and the maxima of our cluster.

cluster	maximium	x	y	z	Network	Task acitvation
1	2	16	-75	-35	Frontoparietal network	Language
	Term	z-Score				
	Lanuage comprehension	6.3				
	Linguistic	6.22				
	Comprehension	4.94				
	Paired	4.35				
	Spoken	4.31				
	Reading	4.12				
2	1	53	-59	-30	Default network	-
	Term	z-Score				
	memory tasks	5.35				
	face	5.12				
	unimodal	5.05				

	blind	4.98					
	shapes	4.94					
	noxious	4.8					
	compensation	4.73					
3	3		13	-68	-22	Ventral attention network	Motor task
	Term	z-Score					
	motor	6.43					
	supplementary	5.28					
	production	5					
	movements	4.69					
	serial	4.62					
	supplementary motor	4.61					
	reading	4.52					
	verbal working	4.47					
	musical	4.42					

	pseudowords	4.11					
	virtual	3.92					
	movement	3.87					
	phonological	3.75					
	finger	3.65					
	auditory	3.5					
3	5	2	-63	-26		Ventral attention network	-
	Term	z-Score					
	heart rate	5.67					
	tapping	5.25					
	force	5.03					
	flexibility	4.51					
	primary motor	4.33					
	hand	4.12					
	movement	4.11					

motor	4.06
timing	4
primary	3.59

The z-score shows the degree to which each voxel is activated in studies that use the term

Table 2 supp: Results of the analysis of the neurosynth.org-database and the maxima of our cluster.
